# Comparison between Internalizing Anti-HER2 mAbs and Non-Internalizing Anti-CEA mAbs in Alpha-Radioimmunotherapy of Small Volume Peritoneal Carcinomatosis Using ^2^ ^1^ ^2^Pb

**DOI:** 10.1371/journal.pone.0069613

**Published:** 2013-07-29

**Authors:** Vincent Boudousq, Laure Bobyk, Muriel Busson, Véronique Garambois, Marta Jarlier, Paraskevi Charalambatou, André Pèlegrin, Salomé Paillas, Nicolas Chouin, François Quenet, Patrick Maquaire, Julien Torgue, Isabelle Navarro-Teulon, Jean-Pierre Pouget

**Affiliations:** 1 Institut de Recherche en Cancérologie de Montpellier, Institut National de la Santé et de la Recherche Médicale U896, Université Montpellier, Montpellier, France; 2 Service de Médecine Nucléaire, Centre Hospitalier Universitaire de Nîmes, Nîmes, France; 3 Institut régional du Cancer de Montpellier, Montpellier, France; 4 Oniris, Amaroc, Nantes, France; 5 AREVA NC, Beaumont-Hague, France; 6 AREVA Med LLC, Bethesda, Maryland, United States of America; Van Andel Institute, United States of America

## Abstract

**Background and Purpose:**

We assessed the contribution of antibody internalization in the efficacy and toxicity of intraperitoneal α-radioimmunotherapy (RIT) of small volume carcinomatosis using ^212^Pb-labeled monoclonal antibodies (mAbs) that target HER2 (internalizing) or CEA (non-internalizing) receptors.

**Materials and Methods:**

Athymic nude mice bearing 2–3 mm intraperitoneal tumor xenografts were intraperitoneally injected with similar activities (370, 740 and 1480 kBq; 37 MBq/mg) of ^212^Pb-labeled 35A7 (anti-CEA), trastuzumab (anti-HER2) or PX (non-specific) mAbs, or with equivalent amounts of unlabeled mAbs, or with NaCl. Tumor volume was monitored by bioluminescence and survival was reported. Hematologic toxicity and body weight were assessed. Biodistribution of ^212^Pb-labeled mAbs and absorbed dose-effect relationships using MIRD formalism were established.

**Results:**

Transient hematological toxicity, as revealed by white blood cells and platelets numbering, was reported in mice treated with the highest activities of ^212^Pb-labeled mAbs. The median survival (MS) was significantly higher in mice injected with 1.48 MBq of^ 212^Pb-35A7 (non-internalizing mAbs) (MS = 94 days) than in animals treated with the same activity of ^212^Pb-PX mAbs or with NaCl (MS = 18 days). MS was even not reached after 130 days when follow-up was discontinued in mice treated with 1.48 MBq of ^212^Pb-trastuzumab. The later efficacy was unexpected since final absorbed dose resulting from injection of 1.48 MBq, was higher for ^212^Pb-35A7 (35.5 Gy) than for ^212^Pb-trastuzumab (27.6 Gy). These results also highlight the lack of absorbed dose-effect relationship when mean absorbed dose was calculated using MIRD formalism and the requirement to perform small-scale dosimetry.

**Conclusions:**

These data indicate that it might be an advantage of using internalizing anti-HER2 compared with non-internalizing anti-CEA ^212^Pb-labeled mAbs in the therapy of small volume xenograft tumors. They support clinical investigations of ^212^Pb-mAbs RIT as an adjuvant treatment after cytoreductive surgery in patients with peritoneal carcinomatosis.

## Introduction

Peritoneal carcinomatosis is relatively common in gynecological or digestive cancers or primary peritoneal malignancies, such as mesothelioma or peritoneal serous carcinoma [1]. The combination of cytoreductive surgery to treat the visible disease, and hyperthermic intraperitoneal chemotherapy (HIPEC) can improve the patients’ median survival [2], [3], [4], [5], [6]. Nevertheless, this approach is associated with high post-operative morbidity (30–50%) and mortality (4%) due to surgery complications and/or chemotherapy side effects [7], [8].

Several studies in rats have demonstrated that radioimmunotherapy (RIT) could be an alternative approach to HIPEC [9], [10], [11], [12]. However, the results of the only phase III clinical trial on intraperitoneal RIT for ovarian cancer by injection of ^90^Y-HMFG1 monoclonal antibodies (mAbs) were rather unsatisfactory [13], possibly due to the low absorbed doses to the tumors and high incidence of extraperitoneal disease recurrence [14]. The choice of ^90^Y may be questionable for RIT of small volume tumors because the emitted β particles have a long range in matter (0.05–12 mm) and thus they may cause bone marrow toxicity due to non-specific cross fire irradiation. Moreover, as they have very low linear energy transfer (LET = 0.2 keV/µm) they are poorly cytotoxic per unit dose.

Conversely, alpha particles constitute attractive candidates for RIT of single cells or small volume tumors (for reviews [15], [16] ) because they have shorter path length (40–100 µm) and higher LET (50–270 keV/µm) than compared to beta particles and thus they are highly deleterious locally. Moreover, new *in vivo* nanogenerators of alpha radionuclides, such as ^225^Ac/^213^Bi or ^212^Pb/^212^Bi that generate ^213^Bi and ^212^Bi respectively, and new chelating agents that improve the radionuclide-mAb complex stability have improved the availability of alpha particle emitting isotopes for clinical RIT (for review [17]).

Alpha particle emitters, such as ^213^Bi, ^211^At and ^212^Bi (generated from ^212^Pb), have been coupled to monoclonal antibodies, peptides or liposomes for treating leukemia [18], [19], breast [20], [21], prostate [22], [23], [24], ovarian [25], [26], [27], [28], [29], colorectal [30], [31], [32] and bladder [33] cancers in mice. Most of the preclinical studies on RIT with ^212^Pb [30], [31], [34], [35], [36] and the ongoing clinical phase I study in the USA have targeted the human epidermal growth factor receptor 2 (HER2). As anti-HER2 mAbs are internalized in the cytoplasm after receptor binding (for review [37]), ^212^Pb-mAb internalization could contribute to RIT efficacy and toxicity. Indeed, internalization may be associated with high radioactivity uptake via cell surface receptor recycling and it may also help retaining radioisotope daughters (including, for ^212^Pb, the two alpha emitters ^212^Bi and ^210^Po, and the beta emitter ^208^Tl) within the cytoplasm of targeted cells. Some have suggested that,^212^Pb-mAb internalization and the subsequent acidic catalysis within lysosomes may lead to the dissociation of the radio-metal from the chelator and to the release of isotopes from the targeted cells that may produce toxic effects, such as bone toxicity [38].

Therefore, the aim of our work was to compare the efficacy and toxicity of non-internalizing ^212^Pb-35A7 (anti-carcinoembryonic antigen, CEA) mAbs, which mostly remain at the cell surface, and of internalizing ^212^Pb-trastuzumab (anti-HER2) mAbs in RIT of small volume peritoneal tumors that express CEA (high level) and HER2 (lower level) receptors.

## Materials and Methods

### Cell Line and mAbs

HER2-positive vulvar squamous carcinoma A-431 cells obtained from ATCC were transfected with constructs encoding CEA and luciferase [12]. Cells were grown in Dulbecco’s Modified Eagle Medium supplemented with 10% fetal calf serum, 1% penicillin/streptomycin and 1% geneticin at 37°C in a humidified atmosphere containing 5% CO_2_. The IgG1k 35A7 mAb against the CEA Gold 2 epitope was obtained from hybridoma kindly provided by Dr J-P Mach, Lausanne, Switzerland [39] and the anti-HER2 mAb trastuzumab (Herceptin®, Genentech, San Francisco, CA) was also used. The non-specific PX IgG1 mAb was obtained from the ATCC mouse hybridoma P3X63Ag8 [40] and was used for control experiments. The 35A7 and PX mAbs were purified from mouse hybridoma ascitic fluids by ammonium sulfate precipitation followed by ion exchange chromatography on DE52 cellulose (Whatman, Balston, UK). Affinity of 35A7 for CEA is 9.7×10^−8^ M [41] while affinity of trastuzumab for HER2 is 0.1×10^−9^ M [42].

### CEA and HER2 Expression Levels

500×10^4^ A-431 cells were incubated with 20 µg/ml 35A7 or trastuzumab for 1.5 h, then washed twice with PBS before incubation with 5.4 µg/ml anti-mouse IgG-FITC antibody produced in goat (F2653, Sigma-Aldrich, St. Louis, MO, USA) or 7.6 µg/ml anti-human IgG-FITC antibody produced in goat (F9512 Sigma-Aldrich, St. Louis, MO, USA). We determined the number of fluorophores per antibody by measuring optical density at both 280 nm (mAb absorption) and 495 nm (FITC absorption) for given concentration of mAb and found 7.5 FITC per anti-35A7 and 13.1 FITC per anti-trastuzumab mAbs. Samples were analyzed with a Cytomics FC 500-MCL Flow Cytometer (Beckman Coulter, Roissy, France) by recording 5,000 events to analyze CEA/HER2 expression using WinMDI software. Control groups consisted of cells incubated with the secondary antibody only.

In addition, the number of HER2 and CEA receptors at the cell surface has also been determined using two *in vitro* kit assays (Qifikit®, Dako, France; CellQuant Calibrator®, Biocytex, France).

### Immunofluorescence Assays

For immunofluorescence assays, 4×10^3^ A-431 cells were seeded on coverslip. After 2 days, they were incubated with 20 µg/mL 35A7, 35A7 conjugated with the bifunctional chelating agent 1,4,7,10-Tetra-(2-Carbamoyl Methyl)-Cyclododecane (35A7-TCMC), Trastuzumab, Trastuzumab-TCMC or PX mAbs at 4°C or at 37°C for 1 h, then washed twice with PBS and fixed in 3.7% formaldehyde/PBS for 30 min followed by 30-second permeabilization in acetone at –20°C. Cells were washed twice with PBS and incubated with PBS-BSA (1 mg/ml) for 1 h and then in the dark with an FITC-labeled goat anti-mouse Ig (Sigma) in PBS-BSA (1 mg/ml) for 1 h. Cells were washed three times with PBS-BSA and once with PBS and then incubated with 50 µl 4,6 diaminido-2-phenylindole dihydrochloride (DAPI, Sigma, Chemical Co.) for 15 minutes, washed once in PBS and mounted in Vectashield®.

### Conjugation and Radiolabeling

Trastuzumab, 37A7 or PX were conjugated with TCMC (Macrocyclics, Dallas, TX, USA) using a 12-fold molar excess of ligand to mAb as described in [43]. TCMC was chosen based on its stability at low pH, because DOTA undergoes acidic catalysis within lysosomes after internalization and this can dissociate the radio-metal from the chelator, leading to release of isotopes and toxicity [38], [44], [45]. The mAb final concentration was quantified using the BCA Protein Assay Reagent (Pierce, Netherlands). The number of TCMC molecules linked to the mAbs was determined using a spectrophotometry assay based on the titration of the lead-Arsenazo(III) complex [46] and was about 8–10 TCMC/mAb. Trastuzumab-TCMC was from AREVA Med LLC (Bethesda, MD, USA).

The ^224^Ra/^212^Pb generators were provided by AREVA Med SAS (Bessines-sur-Gartempes, Haute-Vienne, France) and radiolabeling with ^212^Pb was performed as described by Dong [47]. Then, 1 mg mAb-TCMC was incubated with 37 MBq ^212^Pb at 37°C for 1 hour and the reaction quenched with 4 µL 0.1 M EDTA. Specific activities were generally around 37 MBq/mg for the three mAbs. The labeling yield (ratio ^212^Pb/^212^Pb-mAbs) was assayed using SG-ITLC 10-cm strips (Gelman Sciences, Ann Arbor, MI USA) developed in 0.15 M NH4OAc buffer, pH 4.0. ^212^Pb-mAbs were retained at the origin whereas ^212^Pb acetate migrated with the solvent front. The strips were dried, cut into 1 cm segments, and counted in a gamma-counter (Hewlett Packard, Palo Alto Instrument, Ca, USA). It was generally <2%. After conjugation, the ITLC analysis demonstrates the absence of remaining unconjugated TCMC post diafiltration, therefore the arsenazo assay in combination with the protein quantification is sufficient to estimate the mole ratio of chelate to antibody. Additionally, since the chelation is performed at a predefined ratio of 1 mg per 37 MBq, the ITLC post chelation with ^212^Pb demonstrates that we consistently have a high labeling yield as well as a consistent recovery yield on the desalting column.

Immunoreactivity of ^212^Pb-mAbs against CEA or HER2 was assessed *in vitro* by direct binding assays using sepharose activated beads (GE Healthcare) coated with human recombinant CEA and HER2. The binding percentage was determined by measuring the antigen-bound radioactivity after overnight incubation followed by 2 washes with phosphate-buffered saline. It was shown to range from 70% to 80%.

### Animal Model

Swiss nude mice (7 week/old females) from Charles River were acclimated for 1 week before experimental use. They were housed at 22°C and 55% humidity with a light-dark cycle of 12 h. Food and water were available ad libitum. Body weight was determined weekly and the mice were clinically examined throughout the study. All animal experiments were performed in compliance with the guidelines of the French government and the INSERM standards for experimental animal studies (agreement B34-172-27). They were approved by the local ethic committee of “Institut de Recherche en Cancérologie de Montpellier” (IRCM/INSERM) and by the Ethic Committee of Languedoc Roussillon (CEEA LR France n° 36) for animal experiments under the number 2012–50.

### Radioimmunotherapy Experiments

Mice were intraperitoneally (ip) grafted with 0.7×10^6^ A-431 cells suspended in 0.3 mL DMEM. Three days post-graft, tumor growth was determined by bioluminescence imaging to segregate mice in homogeneous groups. The following day, mice received a single ip injection of ^212^Pb-35A7, ^212^Pb-trastuzumab or ^212^Pb-PX mAbs. Three different activities were tested: 0.37 MBq (37 MBq/mg) (n = 12, 6 and 4 mice for each mAb), 0.74 MBq (37 MBq/mg) (n = 14, 7 and 6 mice) and 1.48 MBq (37 MBq/mg) (n = 10, 7 and 6 mice). The control groups received one ip injection of NaCl (n = 8) or 40 µg unlabeled 35A7 (n = 8) or trastuzumab (n = 10).

### Tumor Growth Follow-up by Bioluminescence Imaging

**Figure 1:**
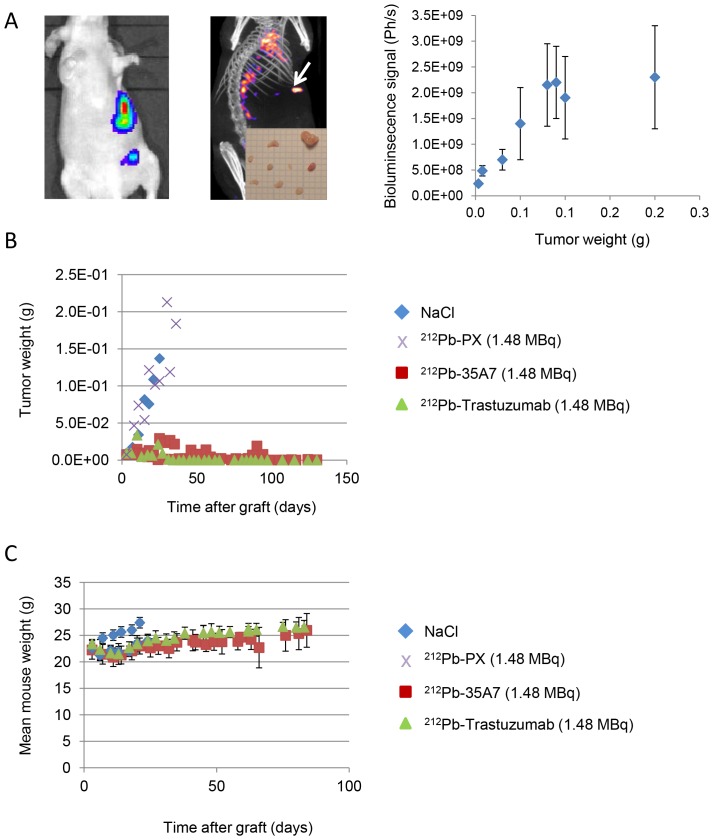
Follow-up of tumor growth and animal weight. (A) Tumor growth was determined by bioluminescence (left) and SPECT-CT imaging (middle). Only the biggest tumor nodules (arrow) could be detected by SPECT-CT imaging at day 1 after injection of 185 MBq ^125^I-35A7. Bioluminescence signal (photons/s) was calibrated as a function of the tumor mass and showed a linear relationship for tumors between 0.01 and 0.08 g (right). Calibration curve equation was y (g) = photons/s×5.0 10^−11^. For larger tumors, signal was saturating. (B) The bioluminescence signal was converted into tumor weight (g) using the calibration curve and plotted versus time post-graft. (C) The mean mouse weight in each treatment group was determined weekly.

Tumor growth was followed weekly by *in vivo* bioluminescence imaging after ip injection of 200 µL luciferin (0.1 mg luciferin/g) as described above [12]. For this purpose, we previously calibrated the bioluminescence signal (photons/s) as a function of tumor weight (g) as described in [12] and reported in Figure 1A. Typically, mice bearing intraperitoneal A-431 tumors xenografts were imaged and next sacrificed for collection and measurement of tumor nodules. The sum of nodules masses per mice was calculated and correlated to the bioluminescence signal. We found a good linearity between the bioluminescence signal and the tumor weight for tumor weight between 0.01 g and 0.08 g. For larger tumors, the dose–response relationship was next saturated, and tumor size was, therefore, underestimated. Mice were sacrificed by CO_2_ asphyxiation, when the bioluminescence signal reached 2.0×10^9^ photons/s for two consecutive times (weekly measurements). Dissection revealed that the real tumor weight was then 2–3×10^−1^ g.

Hematologic toxicity was evaluated using the scil Vet abc system (SCIL Animal Care Co.) and animal weight was determined weekly.

### Biodistribution Experiments and SPECT-CT Imaging

To assess the biodistribution of ^212^Pb-mAbs, mice were xenografted with A-431 cells as described above. Four days later, mice were ip injected with 0.37 MBq (37 MBq/mg) ^212^Pb-35A7 or ^212^Pb-trastuzumab and 30 µg of the relevant unlabeled mAb. At each time point (1, 6, 11, 22, 33 and 44 h after injection), 3–4 animals/group were anesthetized, bled and dissected. The uptake of radioactivity (UOR_Biodis_) of tumor nodules and organs was measured using a γ-counter. The percentage of injected activity per gram of tissue (%IA/g) was then calculated as described in Santoro *et al.* [12].

Due to lack of detection sensitivity, whole-body SPECT/CT images using ^212^Pb-mAbs could not be acquired even for the highest (1.48 MBq) injected activities. Tumors were thus imaged at various times (24 h, 48 h) following ip injection of 18.5 MBq ^125^I-mAbs using a 4-head multiplexing multipinhole NanoSPECT camera (Bioscan Inc.).

### Uptake of Radioactivity per Organ and Tumor, Dosimetry

The uptake of radioactivity (UOR) per tissue (kBq) in RIT experiments (UOR_RIT_) was extrapolated from biodistribution experiments by multiplying UOR_Biodis_ by the ratio between the highest activity used in RIT and biodistribution experiments, namely 4.
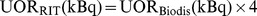



We thus considered that the weight of healthy tissues did not change during the study and that it did not differ between RIT and biodistribution experimental conditions. However, during the two days following the injection of radiolabeled mAbs, tumor weight was measured in biodistribution experiments and found to be slightly higher than compared to RIT experiments at 1.48 MBq of ^212^Pb-mAbs because of the lower activity injected. Tumor weight in RIT experiments was determined from the bioluminescence signal using calibration curves and was 9×10^−3^ g in both ^212^Pb-35A7 and ^212^Pb-Trastuzumab groups. Then, for tumors, UOR_RIT_ was calculated by multiplying UOR_Biodis_ per gram of tumor by the measured tumor weight in RIT conditions and next by the ratio between the highest activity used in RIT and biodistribution experiments, namely 4:
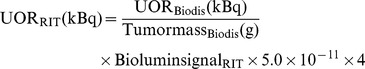



This approach was supported by the finding that UOR_Biodis_ increased linearly with tumor weight and was validated in [12]. The cumulated activity per tissue (Ã_rs_), was next calculated by measuring the area under the UOR_RIT_ curves.

**Figure 2:**
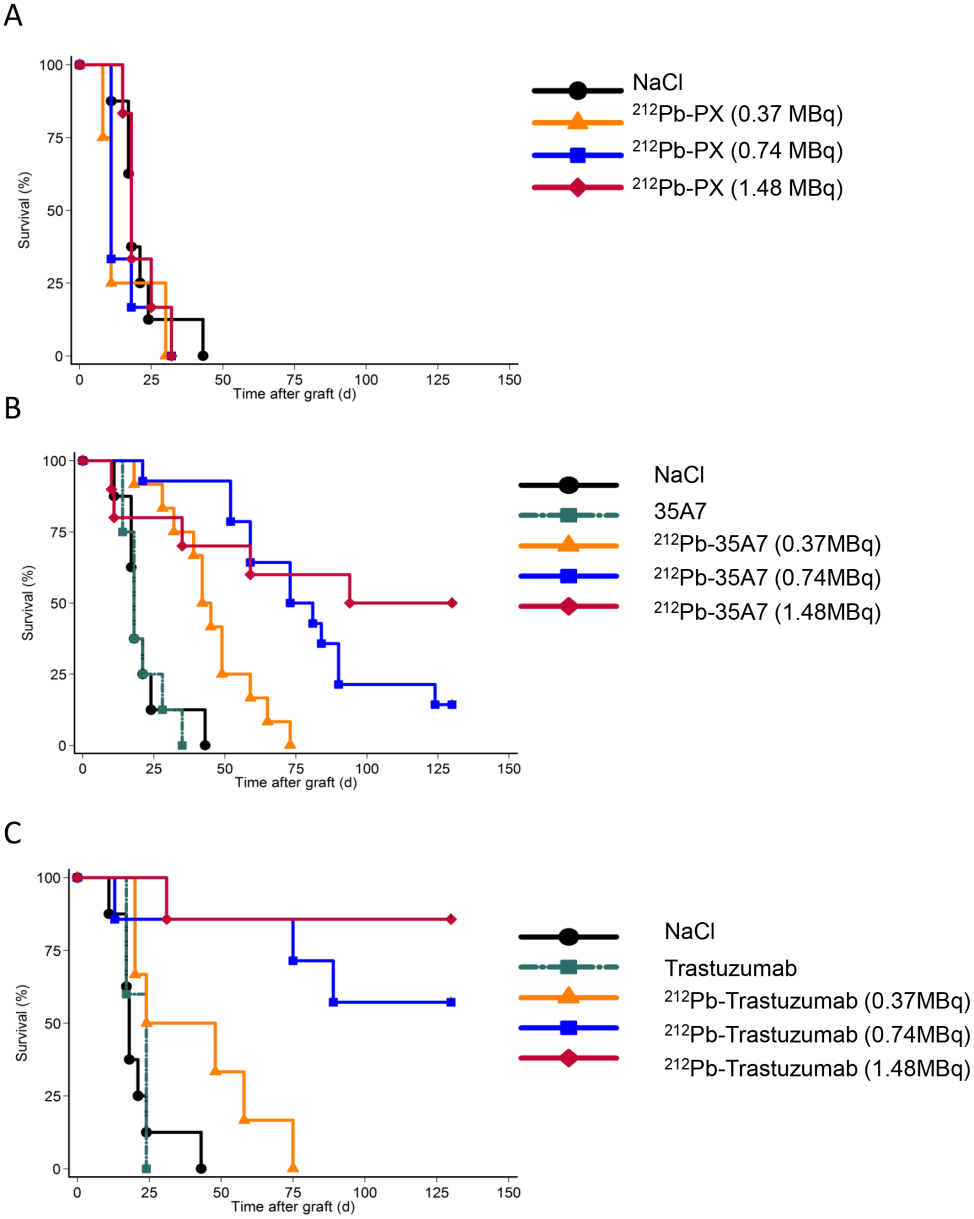
Survival curves. Nude mice xenografted with A-431 tumor cells were i.p. injected with (A) NaCl, the irrelevant ^212^Pb-PX, (B) unlabeled (40 µg) or ^212^Pb-labeled 35A7 (anti-CEA), or (C) unlabeled (40 µg) or ^212^Pb-labeled tratsuzumab (anti-HER2). Mice were sacrificed when the bioluminescence signal reached 2.0×10^9^ photons/s and median survival estimated using the Kaplan–Meier method. Censored mice are indicated by vertical bars.

To estimate the mean absorbed dose delivered to the different organs and tumors, we assumed that the energy emitted during the decay of ^212^Pb and its daughters was deposited locally and totally absorbed. This is a relevant assumption for alpha particles and for most of the beta particles emitted within this decay chain. Thus, the mean absorbed dose to organs/tumors was determined by multiplying Ã_rs_ by 8.7 MeV, which corresponds to the overall energy released by the alpha (7.8 MeV) and beta (0.9 MeV) particles emitted during the decay of ^212^Pb, ^212^Po, ^208^Tl [48]. For the mean absorbed dose delivered to tumors, the contribution from free (i.e., unbound) radiolabeled antibodies in the peritoneal cavity was not taken into account as short-ranged alpha particles should irradiate tumors only superficially and since we finally did not observe any therapeutic effect of non-specific ^212^Pb-PX mAb on survival (Figure 2).

In this dosimetric analysis, two cell S-values (S_N←Cy_ and S_N←Cs_) were also determined for A431 cells and for ^212^Pb and its daughters. Only the alpha particle contribution was considered in these calculations and since the tumors were much larger than the path length of the alpha particles, we calculated the mean absorbed dose in the same manner for both mAbs. Calculations were carried out with a validated small-scale dosimetry code [49] and results were compared with the values of the MIRD tables [25].

### Statistical Analysis

Kaplan-Meier survival estimates were calculated from the xenograft date to the date of the event of interest (i.e., bioluminescence of 2×10^9^ photons/s) and compared with the log-rank test. Statistical analyses were performed using STATA 10.0.

## Results

### Tumor Growth and Survival

**Figure 3:**
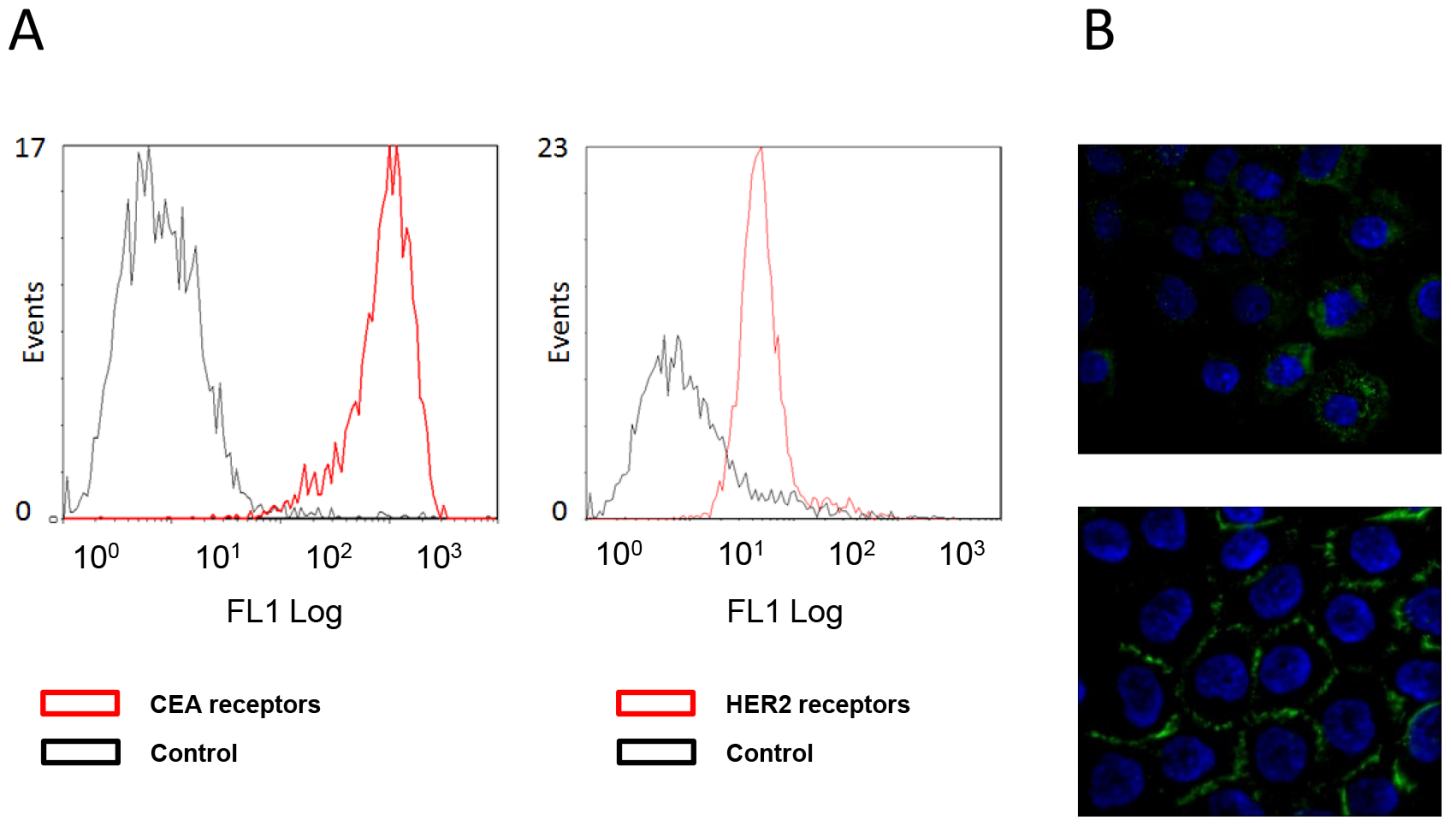
Cell surface receptors and mAbs localization. (A) Flow cytometry analysis of cell surface CEA (left panel) and HER2 (right panel) expression in A-431 cells using the anti-CEA 35A7 and anti-HER2 trastuzumab mAbs, respectively. Control cells were incubated only with the FITC-labelled secondary antibody. (B) Immunofluorescence analysis of the cell localization of trastuzumab (upper panel) and 35A7 (lower panel). Nuclei were stained with Hoechst.

Using flow cytometry analysis, we determined a higher signal of fluorescence for CEA than for HER2 receptors in A-431 cells (Figure 3A) though the number of FITC per anti- 35A7 mAb was lower than the number of FITC per anti-trastuzumab mAb and that the concentration of FITC-mAb was higher for anti-Trastuzumab. However, if affinity of 35A7 for CEA was greater than affinity of trastuzumab for HER2, we couldn’t accede to affinity value of both types of FITC-mAbs for their epitopes. Therefore, we confirmed these data by using *in vitro* kit assays (Qifikit®, Dako, France; CellQuant Calibrator®, Biocytex, France), which similarly indicated that 16±5×10^3^ and 200±35×10^3^ HER2 and CEA receptors, respectively, were expressed at the surface of A-431 cells.

We also confirmed that anti-HER2 (trastuzumab) mAbs, are internalized after receptor binding, while anti-CEA (35A7) ^212^Pb-mAbs, remain at the cell surface (Figure 3B). Tumor growth was evaluated by bioluminescence imaging (Figure 1A, left panel) using the calibration curve described in Materials and Methods. At day 4 post-graft, the mean tumor weight (about 0.01 g) was distributed among 7.2±4.8 nodules/mouse (Figure 1A, middle panel). Only the biggest tumor nodules were detected by SPECT-CT imaging at day 1 or 2 after ip injection of ^125^I-mAbs (Figure 1A, middle panel). No significant difference in tumor growth was observed in mice treated with unlabeled 35A7 or Trastuzumab, ^212^Pb-PX (non-specific mAb) mAbs or NaCl (Figure 1B); conversely, animals treated with ^212^Pb-35A7 or ^212^Pb-Trastuzumab labeled mAbs showed a strong delay in tumor growth. Similarly, no significant difference was observed in the median survival (MS) of mice injected with NaCl (MS = 18 days), unlabeled 35A7 (MS = 18) (p = 0.94) or trastuzumab (MS = 24) (p = 0.45) or different activities of ^212^Pb-PX mAb (0.37 MBq: MS = 11 days, p = 0.37; 0.74 MBq: MS = 11 days, p = 0.32; 1.48 MBq: MS = 18 days, p = 0.85) (Figure 2A). Conversely, survival was significantly higher in mice treated with 0.37 (MS = 42 days), 0.74 (MS = 73 days) or 1.48 MBq (MS = 94 days) ^212^Pb-35A7 (Figure 2B). In animals injected with 0.37 MBq ^212^Pb-trastuzumab, MS was 24 days, whereas in mice treated with 0.74 or 1.48 MBq ^212^Pb-trastuzumab, MS was never reached during the study period (130 days post-graft) (Figure 2C). At day 130, very small-volume tumors (<1 mm^3^) were observed in the only surviving animal treated with 0.74 MBq ^212^Pb-35A7, in two of the five surviving mice treated with 1.48 MBq ^212^Pb-35A7 and in two of the six surviving animals that received ^212^Pb-trastuzumab.

### Toxicity of ^212^Pb Radioimmunotherapy

**Figure 4:**
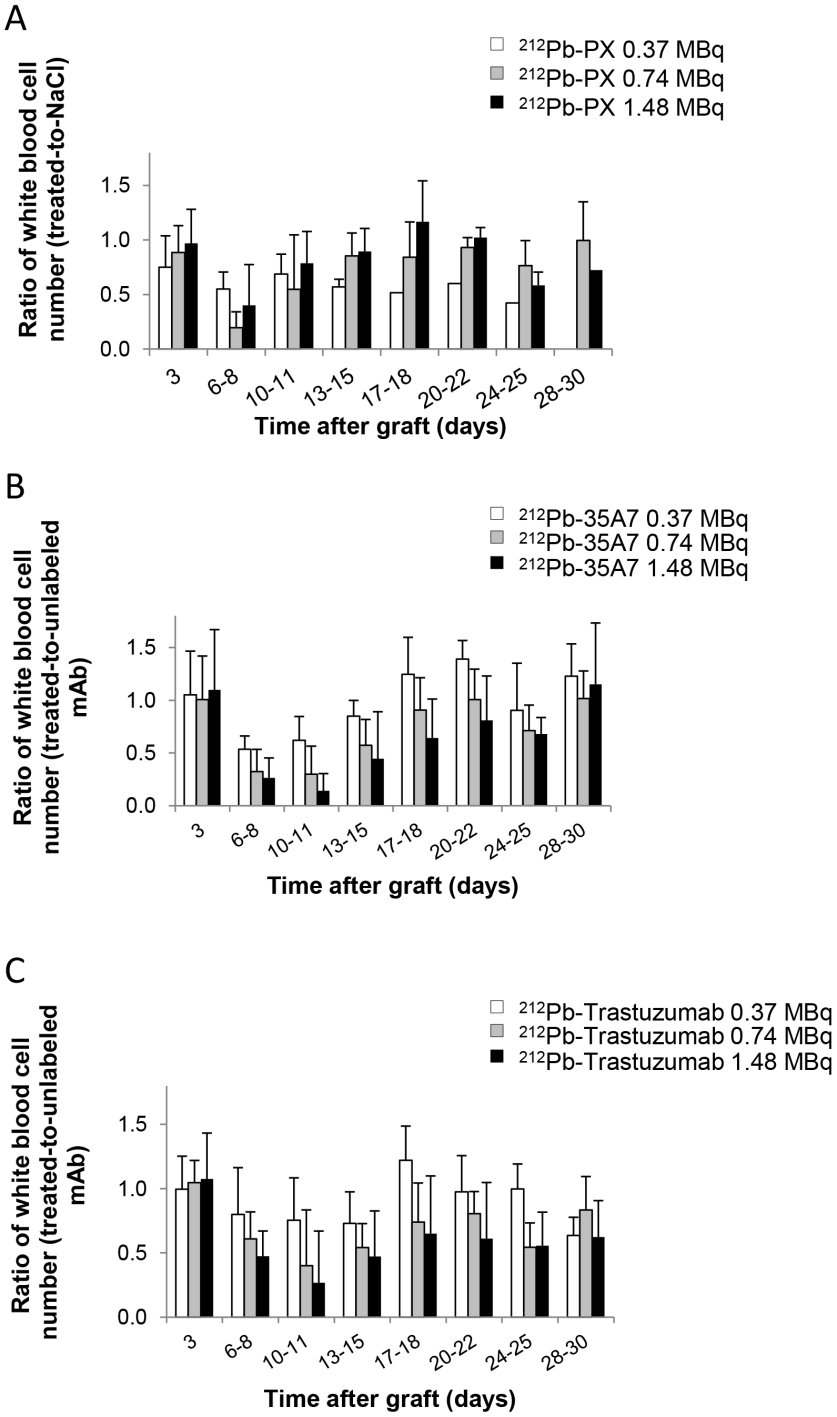
Hematological toxicity. The ratio of white blood cell counts between treated mice and controls was monitored at various times (0–30 d) after treatment with (A) ^212^Pb-PX (non-specific), (B) ^212^Pb-35A7 (anti-CEA) and (C) ^212^Pb-Trastuzumab (anti-HER2 mAbs).

**Figure 5:**
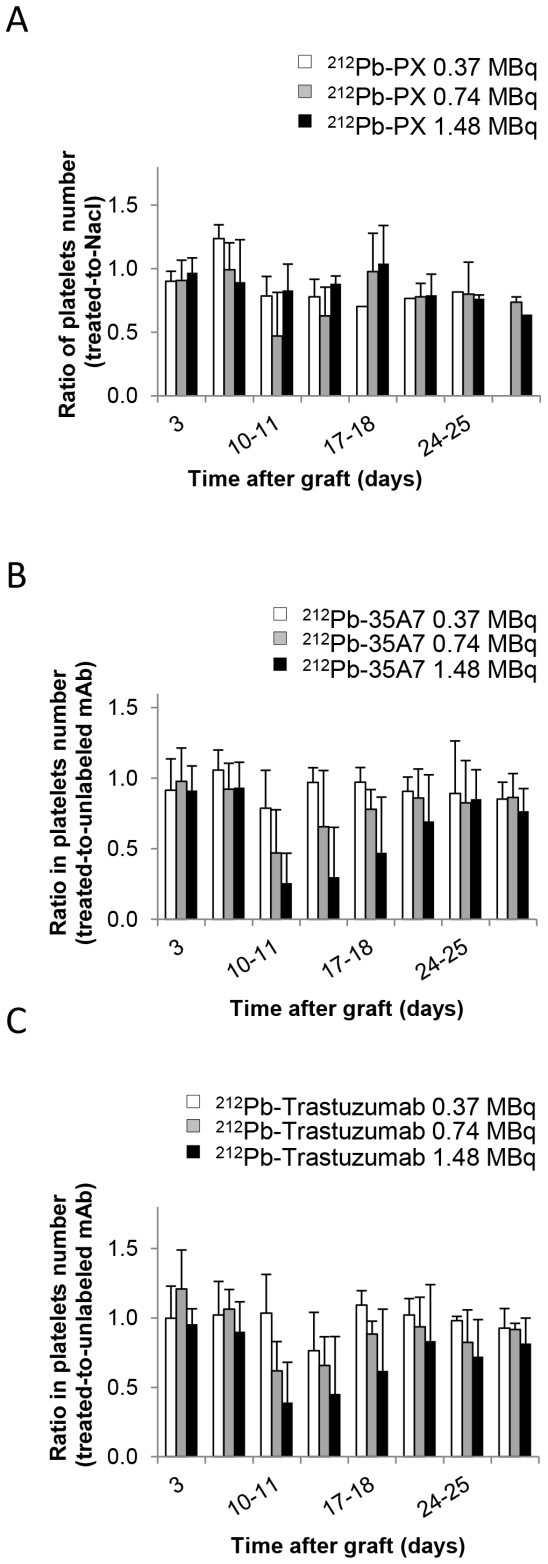
Hematological toxicity. The ratio of platelets number between treated mice and controls was monitored at various times (0–30 d) after treatment with (A) ^212^Pb-PX (non-specific), (B) ^212^Pb-35A7 (anti-CEA) and (C) ^212^Pb-Trastuzumab (anti-HER2 mAbs).

No weight loss was observed in mice treated with ^212^Pb-trastuzumab or ^212^Pb-35A7 (Figure 1C). Hematological toxicity of unlabeled and ^212^Pb-labeled mAbs was assessed by measuring white blood cells (Figure 4) and platelets number (Figure 5) over 30 days following treatment. Results were normalized to those of the NaCl group for PX and to those obtained with unlabeled 35A7 or trastuzumab for ^212^Pb-labeled specific mAbs. All mice treated with ^212^Pb-mAbs showed a transient and activity-dependent hematological toxicity. Reduced number of white blood cells and platelets was also observed following treatment with 0.74 and 1.48 MBq of the two specific ^212^Pb-mAbs.

### Biodistribution of ^212^Pb-labeled mAbs

**Figure 6:**
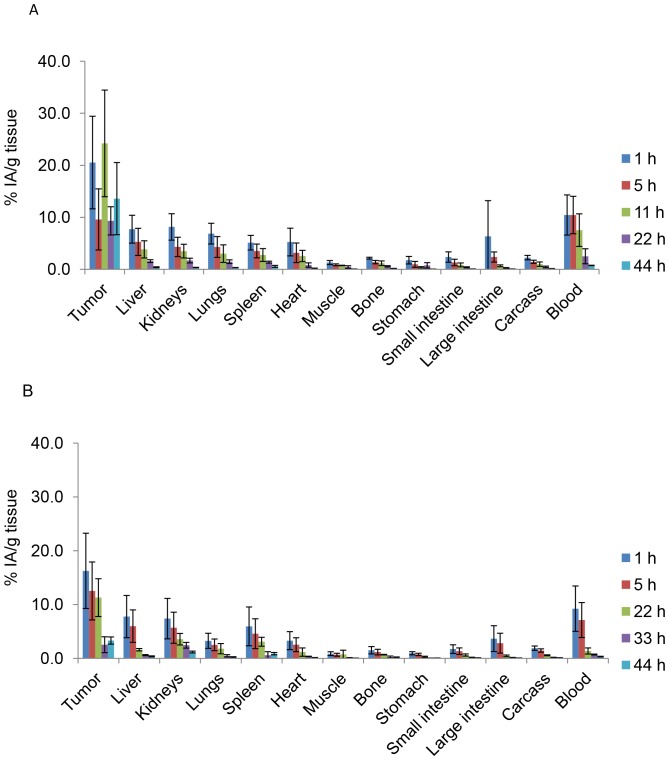
Biodistribution of ^212^Pb-labeled mAbs in nude mice bearing A-431 tumor cell xenografts and injected with (A) ^212^Pb-35A7 (anti-CEA) or (B) ^212^Pb-trastuzumab (anti-HER2). %IA/g (not corrected for decay) was determined in healthy organs and tumors. Four mice were analyzed at each time point.

^212^Pb-mAb biodistribution was determined over 44 h in tumor nodules and healthy tissues and uptake of radioactivity was measured (UOR_Biodis_). Maximal concentrations for tumors were 20.5±8.9 and 16.25±6.9 of the injected activity per gram of tissue (%IA/g) for ^212^Pb-35A7 and ^212^Pb-trastuzumab, respectively. Maximal uptake was measured 1 h after injection for trastuzumab while it was measured at 11 h for 35A7. However, the latter measurement is associated to large error bars and we previously showed that maximal uptake of ^125^I-35A7 in peritoneal tumor was observed 1 h after ip injection [11]. Then, maximal uptake measured 11 h after injection is likely to be overestimated or attached to large uncertainty. Maximal uptake was next measured in blood with 10.4±3.9 and 9.22±4.0%IA/g for ^212^Pb-35A7 (Figure 6A) and ^212^Pb-trastuzumab (Figure 6B), respectively. Next, liver, kidneys and lungs showed the highest values with 7.7±2.7, 8.2±2.5, and 6.9±2.0%IA/g (^212^Pb-35A7, Figure 6A) and 7.7±3.2, 7.3±3.7, and 3.2±1.4%IA/g (^212^Pb-trastuzumab, Figure 6B), respectively.

### Uptake of Radioactivity and Dosimetry

**Figure 7:**
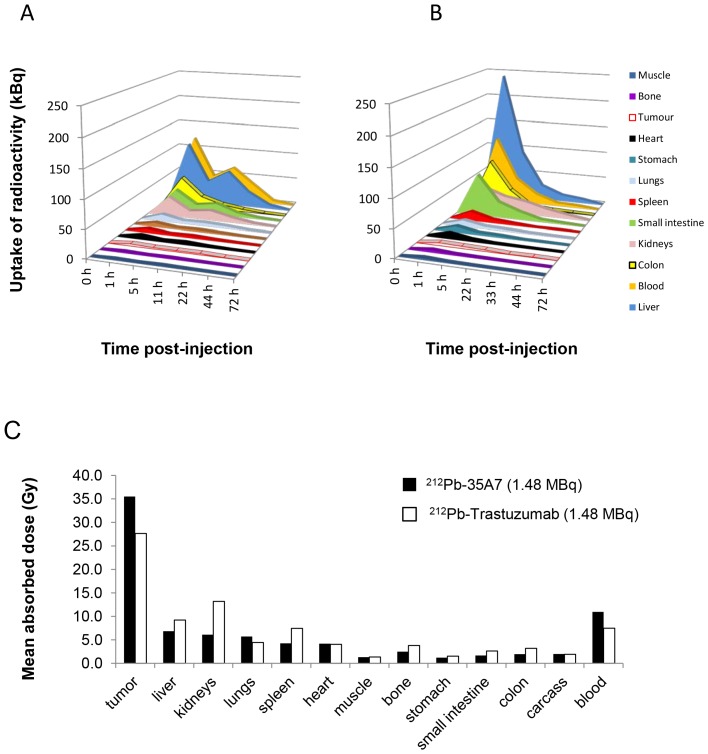
Uptake of radioactivity (UOR_RIT_) per tissue (Bq) was determined using the values obtained during biodistribution experiments (Figure 6). (A) ^212^Pb-35A7 (anti-CEA). (B) ^212^Pb-Trastuzumab (anti-HER2). (C) Mean absorbed doses for ^212^Pb-35A7 and ^212^Pb-Trastuzumab. The total cumulative decay per tissue (Ã_rs_) was calculated from the area under each UOR_RIT_ curve and multiplied by 8.7 MeV (the mean energy emitted at each decay of ^212^Pb and daughters). The ratio between tumor absorbed dose and blood absorbed dose was 3.2 (^212^Pb-35A7) and 3.7 (^212^Pb-Trastuzumab). The tumor-to-organ absorbed doses ratio were 5.2 (liver) and 5.8 (kidney) for ^212^Pb-35A7 and 3.0 (liver) and 2.1 (kidney) for ^212^Pb-Trastuzumab.

As a preliminary step towards the assessment of the absorbed dose, the UOR_Biodis_ values expressed in kBq were multiplied by 4, as described in Materials and Methods, which corresponds to the ratio between the highest activity used in RIT experiments (1.48 MBq) and the activity injected for biodistribution analysis (0.37 MBq) (Figure 7). The uptake of radioactivity during RIT UOR_RIT_ was then obtained and was maximal at 1 h after injection of ^212^Pb-35A7 ranged between 1.46 kBq (bone) and 349.2 kBq (carcass, data not shown), with 3.77 kBq for tumor nodules, 7.29 kBq for spleen, 35.82 kBq for kidneys, 114.4 kBq for liver. For ^212^Pb-Trastuzumab, the maximal UOR_RIT_ ranged between 4.1 kBq (bone) and 310.4 kBq (carcass, data not shown), with 4.98 kBq for tumor nodules, 16.82 kBq for spleen, 240.43 kBq for liver.

The cumulative activity per tissue Ã_rs_ was determined by calculating the area under the curves in Figures 7A and 7B. The mean absorbed dose per organ was calculated by multiplying Ã_rs_ by the total amount of energy emitted by ^212^Pb and its daughters (that were assumed to be deposited locally and totally absorbed) and by dividing the result by the organ mass. The mean absorbed doses were at the highest activity, 35.5 Gy for ^212^Pb-35A7 and 27.6 Gy for ^212^Pb-Trastuzumab in tumors and 10.9 and 7.2 Gy, respectively, in blood (Figure 7C). ^212^Pb-35A7 mAbs were less efficient than ^212^Pb-trastuzumab, although the dose absorbed by the tumor was higher for ^212^Pb-35A7.

Thus the ratio between tumor absorbed dose and blood absorbed dose was 3.2 (^212^Pb-35A7) and 3.7 (^212^Pb-Trastuzumab). The tumor-to-organ absorbed doses ratio were 5.2 (liver) and 5.8 (kidney) for ^212^Pb-35A7 and 3.0 (liver) and 2.1 (kidney) for ^212^Pb-Trastuzumab (Figure 7C).

## Discussion

Here, we investigated the efficacy and toxicity of ^212^Pb-labeled anti-CEA (non-internalizing; 35A7) and anti-HER2 (internalizing; trastuzumab) mAbs in RIT of small tumors. To this aim, we xenografted nude mice with carcinoma A-431 cells that express both CEA (high level) and HER2 (lower level) receptors.

We then injected them with ^212^Pb-35A7 and ^212^Pb-trastuzumab mAbs that were previously coupled to TCMC to provide resistance to intralysosomal acid catalysis [44], seen by others, with internalized ^212^Pb-DOTA-mAb complexes leading to release and accumulation of ^212^Pb in bone and causing bone marrow toxicity [45]. One hour after injection, the bone uptakes of^ 212^Pb-trastuzumab (1.5±0.6%) and ^212^Pb-35A7 (2.2±0.2%) were comparable. However, the bone absorbed dose calculated over 72 h was slightly higher for ^212^Pb-trastuzumab (3.8 Gy) than for ^212^Pb-35A7 (2.5 Gy). The higher bone absorbed dose of ^212^Pb-trastuzumab was not associated with higher hematological toxicity as the WBC and platelet count nadir were more marked in mice treated with ^212^Pb-35A7.

Although the expression level of HER2 was lower than that of CEA receptors, the cumulative UOR_RIT_ by tumors was in the same range for both targeting models (2.8×10^8^ for ^212^Pb-35A7 and 1.9×10^8^ Bq.s for ^212^Pb-trastuzumab). This suggests that the lower HER2 expression was partly compensated by HER2 recycling at the cell surface, where receptors could be targeted again by ^212^Pb-mAbs. Nevertheless, the final tumor mean absorbed dose was still higher for ^212^Pb-35A7 (35.5 Gy) than for ^212^Pb-trastuzumab (27.6 Gy).

Calculation of the median survival showed a dose-dependent MS increase in mice treated with increasing activities of the two mAbs. However, if our study showed that the blood absorbed dose, higher for ^212^Pb-35A7 (10.9 Gy) than for ^212^Pb-trastuzumab (7.5 Gy) mAbs, was in agreement with the higher hematological toxicity of ^212^Pb-35A7 mAb (Figure 4), it also highlighted the lack of absorbed dose-effect relationship between tumor absorbed dose and survival because ^212^Pb-trastuzumab mAbs were more efficient than ^212^Pb-35A7 mAbs.

Though the range of absorbed doses that we found was quite in agreement with data from literature [50], a possible explanation for this lack of correlation relies on the approaches and assumptions done to calculate the mean absorbed dose. Alpha-particle dosimetry has been reviewed extensively in MIRD22 pamphlet abridged in [51]. First, we took into account the mean energy (8.7 MeV) released by alpha and beta particles that are emitted during the decay of ^212^Pb and its daughters, but not the emitter’s subcellular localization (cell surface or cytoplasm). Nevertheless, from the calculations of the S-values for A-431 cells (cell and nucleus diameters: about 14±4.5 and 9.2±1.9 µm [52]), we derived that, when considering alpha-emission only, every decay occurring in the cytoplasm delivered a 1.5 times higher dose to cells than a decay occurring at the cell surface (for the whole chain of decay S_N←Cy_ = 3.06×10^−2^ Gy/Bq.s and S_N←Cs_ = 1.98×10^−2^ Gy/Bq.s). If subcellular localization is an important parameter when evaluating the efficacy in isolated tumor cells, or very small micro-metastases, its influence is negligible in our work since tumors of a few millimeters in diameter are studied. Specifically, by using a validated small-scale dosimetry code [49], we found that the mean absorbed dose delivered to one A-431 cell surrounded by similar cells (a cell packing of 0.74 was assumed in this model and one ^212^Pb atom attached to each cell) was the same (0.64 Gy) with internalizing and non-internalizing radiolabeled vectors. This result could be explained by the fact that, in 1–2 mm (or larger tumors) tumors, 95% of the received dose is due to cross-fire. This observation also invalidates the hypothesis that the release from TCMC in the extracellular space of the short lived ^212^Po (T_1/2phys_ = 2.9 10^−7 ^s) might reduce the mean tumour absorbed dose when non-internalizing mAbs are used.

Secondly, we assumed that for both ^212^Pb-mAbs S-values, ^212^Pb was in equilibrium with its daughters. This is true for analysis times longer than 5 hours following elution of the radiolabeled mAbs through the gel size exclusion column after radiolabeling. However, as the same hypothesis was used for both targeting models, this overestimation of the mean absorbed doses cannot interfere with our conclusions.

Thirdly, the distribution of internalizing and non-internalizing ^212^Pb-mAbs within tumors and subsequent absorbed dose distribution at the organ scale would deserve to be further investigated since mean absorbed dose does not allow taking into account heterogeneity in dose distribution that could be associated to higher therapeutic effects per Gy of internalizing^212^Pb-mAbs.

Finally, the discrepancy between absorbed doses and biological effects could also be due to biological phenomena. For instance, while anti-CEA mAbs do not interact with any identified signaling pathways, unlabeled anti-HER2 mAbs are known to block the cells in G1 phase of the cell cycle and may down regulate HER2 receptors and disruption of receptor dimerization and signaling through the downstream PI3K cascade [37]. Although, the tested amount (40 µg) of unlabeled mAb did not have any direct impact on tumor growth, we cannot exclude any synergetic effect between trastuzumab and ^212^Pb irradiation that may influence the final outcome of therapy. This and the contribution of bystander effects need to be assessed in further studies.

It must also be kept in mind that calculating accurately the uncertainty associated to mean absorbed dose values is a tedious task since it combines several sources of uncertainties: uptake of radioactivity at each time point, final cumulated uptake of radioactivity, tumor and organ masses, and *S*-values. Such uncertainties were not calculated in the present study and statistics about differences between calculated mean absorbed doses could therefore not be established.

Our study confirms the strong efficacy of RIT with ^212^Pb-mAbs in animal models of cancer. When ^212^Pb-mAbs were tested in previous preclinical studies [30], [31], [34], [35], [53], TCMC was used as chelator only in three works [30], [31], [35] and tumors were targeted with trastuzumab. Thus, Milenic *et al.* reported that in mouse models of pancreatic and colorectal peritoneal cancer [30] MS increased from 19 days (sham) to 56.5 days after ip injection of 0.74 MBq ^212^Pb-trastuzumab. However, no difference was observed between mice treated with 0.48 and 0.74 MBq suggesting than the lowest activity could then be used. Tan *et al.* demonstrated that one intravenous injection of 0.74 MBq ^212^Pb-trastuzumab delayed tumor growth without significant toxicity in an orthotopic model of human prostate tumor. In our study, we observed, as mentioned above, an absorbed dose dependent effect for each radiolabeled mAbs considered alone and non-internalizing ^212^Pb-35A7 were shown to be more effective after injection of 0.37 MBq than the 2 other radiolabeled mAbs used at the same activity. Indeed, 0.37 MBq ^212^Pb-trastuzumab (MS = 24 days versus 11 in controls) was less effective than 0.37 MBq ^212^Pb-35A7 (MS = 42 days). This lack of efficacy could be artefactual because the two Kaplan Meyer survival curves were rather similar (Figure 2) with sacrifice of the last mice at day 75 post-graft in both groups.

Moreover, if it was shown in previous studies that high activity (1.48 MBq) of irrelevant ^212^Pb-mAbs was accompanied by significant toxicity [30] and was associated with some therapeutic effects [31], our study indicated that the MS of mice treated with the irrelevant ^212^Pb-PX mAb, unlabeled mAbs or NaCl were not statistically different, demonstrating the lack of effect of non-specific irradiation in this tumor model.

The high efficacy of both anti-HER2 and anti-CEA ^212^Pb-mAbs in our study may be explained by the nature of the tumor cells, or more likely by the tumor volume at the time of treatment. We treated 10 mm^3^ tumors while volumes ranged from 15 mm^3^ [30] to 100 mm^3^ in [35] in previous studies. Our results support the idea that RIT of solid tumors should be dedicated to small volume tumors, such as peritoneal carcinomatosis that can originate from CEA-positive ovarian or digestive tumors. Although anti-HER2 ^212^Pb-mAbs were the most efficient, the MS of mice treated with 1.48 MBq anti-CEA ^212^Pb-mAbs was also strongly improved (94 days versus 18 days for NaCl-treated controls), supporting the hypothesis that intraperitoneal RIT with anti-CEA ^212^Pb-mAbs could be an alternative to HIPEC in digestive cancers expressing CEA.

To date, clinical alpha particle RIT has been investigated in patients with hematological malignancies [54], [55], ovarian [56], melanoma [57] or brain tumors [58]. Our study gives preclinical rationale for the ongoing Phase I study (NCT01384253) as the toxicity was less than previously reported suggesting the potential for dose escalation with acceptable toxicity and a higher therapeutic efficacy.

### Conclusion

We have shown that internalizing anti-HER2 ^212^Pb-mAbs targets antigen expressing xenografts and are more efficient per Gy than non-internalizing anti-CEA ^212^Pb-mAbs in reducing and eradicating tumor growth. Treatment was associated with only transient and tolerable hematologic toxicity. This preclinical data gives support to proceeding with clinical trials with this RIT agent.
